# Texas State Medical Association

**Published:** 1900-06

**Authors:** 


### The Medical Societies.

### TEXAS STATE MEDICAL ASSOCIATION.

### Thirty-second Annual Meeting, Waco, Texas, April 24-28, 1900.—Proceedings (Continued).

Discussion on Dr. H. A. West's paper, "The Maladministration of Medical Affairs in the State of Texas," (published in our May number, which please see).

DR. F. E. DANIEL: The subject which my friend, Dr. West, has presented so well is one of such gigantic proportions that no one present seems to be disposed to tackle it. For my own part, I confess my reluctance to take hold of the matter at all. I will attempt no criticism whatever upon his paper with reference to the quarantine department, but I wish to say that I endorse all that the doctor has said. He has said it in a straightforward and clear manner, and there is no denying the truth of the charges. It is a lamentable condition, and the question is: What is the remedy? These things have been pointed out to our statesmen and governors for twenty years, but we might as well have sung psalms to a dead man. It has done no good. He arraigns the State of Texas in a masterly manner. The indictment is a terrible one. But he might as well arraign the whole United States of America upon this same charge. The health interests of America are shamefully neglected. Our congressmen, our statesmen, our governors, have, as far as I am able to judge, entirely failed to grasp the fundamental meaning of "State medicine." Consequently they can have no conception of its scope. It does not seem to have dawned upon them that it is the highest duty of the State to protect the public health. No effort is being made to protect the public health by any other means than the exclusion of disease which, if proper precautions had been enforced, ought never to have come to our shores. Quarantine ought to be abolished. Diseases which we seek to guard against ought never to be allowed to leave their homes in Rio Janiero, Cuba, and elsewhere. It is humiliating to have to acknowledge that the State of Texas has no board of health. I have frequently been interrogated by United States medical officers and editors of papers, about our "board of health," and I have had to tell them that we have none. We take cognizance of no feature of State sanitation or preventive medicine except as relates to quarantine. The United States government, one of the foremost "powers of the world," has no system of collecting and preserving its vital statistics. Our congressmen and statesmen and governors seem to have entirely failed to grasp the idea that without a record of vital statistics no knowledge of the prevalence of disease and the consequent mortality can be obtained, when the history of sanitation and the history of medicine is teeming with proofs of the good effects of sanitary means instituted in other countries, based upon a study of vital statistics. We have failed to make our legislators or our governors understand that the life of the people in the nation can be lengthened by removing the causes of disease when we have a knowledge of those causes.

Many instances can be cited where the average length of life of a community has been extended by sanitary methods. It is not necessary to cite instances of that kind. For some reason our legislators do not seem to comprehend that the preservation of the public health by every legitimate means in its power is the highest duty of the government and adds greatly to the strength, wealth, and prosperity of the nation more than any other factor. They have failed to perform that duty. Some one has said that every death from a preventable disease is a crime. So it is. The State, or county, or town, has as much right to kill a man with a hatchet or a gun as to kill him with a defective sewer or polluted water. I move that Dr. West's paper be printed in pamphlet form for a "campaign document."

THE CHAIR: I suppose that will come up in regular meeting. This is a section meeting.

DR. R. H. HARRISON: It is not necessary for me to add a word to what has been said on this subject this evening. The exhaustive character of the paper that has been read to this Association, and the remarks of "Brother Daniel" have been sufficient for the present purpose. I move that the paper be referred to a spcial committee for a special report to this Association.

THE CHAIR: Your motion should come up in regular meeting.

DR. J. W. SCOTT: I have nothing particular to say in regard to this paper, except that I agree with everything Dr. West has said. There is no doubt of the fact that it is easier to find fault, and it is easier to tear down, and break up and destroy, than it is to build up, but at the same time there are faults of which no medical man should be guilty, and one of those faults is, that he should not stand still and refuse positively to endeavor to make any progress, especially in the lines of preventive medicine. One of the principal objections I have to the manner in which the State health department has been conducted is this: They have positively refused to have any dealings with any of the other State departments of the South. The other States have held meetings in Atlanta and in New Orleans, but the State health department has always positively refused to take any part in those meetings. In other words, it says: "We have reached perfection. We cannot go any farther. The quarantine system is perfect and we do not propose to take any chances." That is one of the principal objections that I have.

DR. A. NOLTE, of New Orleans: I would merely say that, of course, I do not know the methods of your asylums, etc., but as regards the quarantine portion of the paper I must modestly beg to disagree. Dr. West undobutedly states that which he believes to be true. I only differ from him in his laying the fault upon the health officers of the States. I think it is with the people. I think they are not yet prepared to see any great modification in the quarantine regulations. I believe it, therefore, to be the duty of the medical men here assembled, who are, after all, the educators, to go to their respective counties and talk up this matter of modifying the quarantine regulations. They are undoubtedly rather a hindrance to your commerce, and that means a great deal. I think it is the duty of every medical man here to go to his town and tell the people of these things. The rules adopted by the Atlanta convention, and that applies also to the convention at New Orleans, and somewhat modified by the State of Alabama, are decidedly strict, and yet they allow of freight and commerce. The passenger restrictions, of course, are still on in Alabama, and I am candid enough to believe that they should be.

Dr. I. B. Massey: I am glad to have the privilege of giving my assent to the greater portion of the statements contained in Dr. West's very able paper. The arraignment is terrible, indeed, and I am afraid it is too true in almost every detail. The only point that I wish to make is this: I am afraid that the doctor's paper is pointed in the wrong direction. The efforts of the State Medical Association, as has been mentioned before, for probably twenty years have been directed in an effort to correct these abuses, but I am afraid they have always been pointed in the wrong direction. The attack has been made against the officials who are in power, and whose business it is to execute the laws that are made. The efforts have not been directed against the people who make the laws, nor the people who are behind these lawmakers, and whose views these lawmakers exemplify in the laws that they pass. The laws you find on the statute book of any State are a fair illustration of what the people of that State want. When it comes to such times that the people in a State want a law changed it does not stand very long on the statute book. And an arraignment of the people who have been elected and appointed to execute these laws I think is directed in the wrong direction. The efforts of this Association should be directed to an education of the people up to these things. That is where our efforts should be directed, and when they are directed in that direction and worked on long enough, until this campaign of education is thoroughly complete, then your laws will be changed, and not until then. It seems to me that your legislative committee should have found this out long ago. You have sent committees to the Legislature time and again to try to influence legislative action by the State Legislature, and you have failed every time, as was stated this morning in the able report of the chairman of the legislative committee. This Association has failed year after year, until it looks to me like you would have taken the hint long ago. Begin your campaign of education at home. Educate your people up to what they should have, the laws they should have in force on the statute book, and then those laws will be placed there in short order. An arraignment of this kind of the State officials exhibits a terrible state of affairs, but the people inaugurated it and they are not prepared today for a change, and will not be prepared until this campaign has been opened. We have more smallpox in the State of Texas today than has ever existed, yet in many counties the condition of affairs is absolutely ignored by the county officials and city officials, and I am afraid in some instances those things are being ignored upon the advice of physicians who ought to know better and who do know better. I hope that each and every one of you will return to your home and begin an active campaign of education. Teach your people the evils arising from the present system of quarantine, the necessity for a State health department instead of a quarantine department, and it will only be a very short time until you will find that the legislative enactment of what you want will be a very easy matter to procure.

Prof. Keiller: Most of the management of insane asylums and the whole question of quarantine and State health is going to be improperly managed until these appointments are taken out of the arena of politics, when the State recognizes that there are certain appointments that should be given to the very best men, no matter where they come from, and so long as they are competent they should be allowed to retain their positions, and when they are incompetent they will be dismissed. When they get such men as can properly advise boards whose advice is to be valued, the whole thing will be upon a permanent basis, and there is a possibility of some progress; but so long as State health officers are appointed only for an exceedingly limited time, and are likely to be set aside and replaced by others just as they are getting to know their business, I think very little progress is to be made.

Dr. West: I have very little more to say. I have had my say. One word in regard to this "campaign of education." I drew from Dr. Massey's remarks, and Dr. Nolte's, that perhaps they thought we were going about this matter in the wrong way. Now, it appears to me that if education is wanted, and I am not disposed to dispute that fact, we have got to educate the officials and educate the doctors, too. I have no doubt that I have drawn some attention of the gentlemen here this evening to facts that perhaps you had not thought of before, and the best way and the only way I know of of educating the people is to bring these facts to your attention and let you take action upon them in your official capacity as well, and then let the education go along down the line from the Governor to the voter. I think education is needed all along the line, and I am free to admit that we need it, and we need it very badly. It is to invite an attempt in that direction that has induced me to read this paper.

Dr. M. M. Smith (appointed to "present the subject") addressed the Association as follows:

"THE PUBLIC HEALTH AND THE STATE'S DUTY TO PROTECT IT."

"*Mr. Chairman and Gentlemen of the State Association:*

"I was appointed at the San Antonio meeting to present the subject of public health and hygiene at this meeting. The time that I had set aside for the preparation of a paper suitable for this occasion had to be utilized otherwise; hence I come before you unprepared entirely. However, the importance of the subject demands that I should say something, although Dr. West has 'stolen all of my thunder.' I am very much like the little boy in the country school who got up to say his first Friday afternoon speech. He got up, and thought he had a speech to say, but the teacher who prompted him had all of the work to do. So, on this occasion Dr. Daniel, who will follow me and open the discussion, and who has given many years of study to this question, will furnish you food for thought, and I ask for his words your careful consideration.

"I had selected for the subject of my remarks when I thought of preparing a paper, 'The Public Health and the State's Duty to Protect it.' I believe it is not necessary to go into a definition of what is understood by the public health. The medical profession and even the people at large understand what is meant. I shall, therefore, pass that portion of the question. As to the State's duty to protect the public health, I believe that the establishment of State boards of health in more than thirty of the States of this nation, and the establishment of the Marine Hospital Service of the United States, is sufficient evidence, and will act as a guaranty that it is the duty of the State to protect the public health. I will say, however, in passing, that in the establishment of this government, when the Declaration of Independence was made, and when the thirteen States declared their independence from their home government, in that declaration we find that man possesses certain 'inalienable rights,' among which are 'life, liberty and the pursuit of happiness.' You will see they have placed life first; then follow liberty and the pursuit of happiness. So you see the most important of all questions is the protection of life, or the protection of the public health. It is, therefore, the duty of the State to protect the health of its people in every possible way.

"I will refer very briefly to our present health laws, though Dr. West has already referred to them. We find in looking upon our statutes they have only two provisions, you might say. One is that the State Health Officer shall have the authority and is empowered to appoint analysts and chemists to examine the adulteration of foods and impure products, and shall publish lists of the same whenever he deems it necessary, but shall not expend more than $2000 per year for such work. This has always been a dead letter on our statute book. Whether the State Health Officer has felt it was not necessary, or whether the Legislatures have failed to make suitable appropriations, I cannot say. However, that portion of the law has never been enforced.

The second portion of our health laws refers to the quarantine in this State, and, as has already been mentioned by Dr. West, the present health laws deal solely with quarantine. We have quarantine against yellow fever, smallpox, cholera, and the bubonic plague, and we establish quarantine along the gulf coast and along the Mexican border. This is what the public health system of Texas consists of today. We have made no provision for the protection of the health of the people against, for instance, tuberculosis, from which disease more than one-seventh of the human family die annually. A disease which is scattered broadcast all over the land, and a disease, gentlemen, that were it dealt with properly by every State in this nation for five or ten years, if the people were educated, we could reduce its mortality more than one-half at least. We have made no provision for the protection of life against typhoid fever, nor, in certain sections, against scarlet fever. There are some little provisions made in some localities, but I am speaking now in general terms. In a State that has the largest sea coast to protect in the United States, and the largest land border to guard, and more territory than any other State in the Union, yea, more than all the New England States combined, we depend upon one man to protect the life and health of our entire people. I say it is an outrage that we should be so far behind in this advanced age, when preventive medicine is doing so much to reduce the death rate all over the country and to prolong man's life. I think the proper manner for us to deal with this question is by the establishment of a State Board of Health. I served on a committee from this Association two years ago to present a bill to the State Legislature for the establishment of a State Board of Health, and we, as a committee from the Texas State Medical Association, were almost ignored. We received no recognition, you might say, either from the House or the Senate, further than an invitation to appear before their committee. We really made no progress towards the passage of such a law. We must have in Texas a State Board of Health, with the president of said board to act as our State Health Officer, with authority to declare quarantine whenever the urgency of the case demands, until the board could be called together, when they could act as their wisdom saw proper. Let us by all means have a State Board of Health to prepare rules and send out circulars of instruction all over the State; to appoint health officers in each county; to distribute literature for the education of the people so as to prevent these contagious diseases, and for the accumulation of vital statistics, something so badly needed in this State.

"Again, we need for the protection of the public health of the people of this State a law regulating the practice of medicine. We are having an experience now in the city of San Antonio with a medical college known as the New York Medical College. On the 5th day of December, 1899, the State issued a charter to the New York Medical College, at San Antonio, which was alleged to be an institution for the purpose of educating persons in the branches of medicine and surgery. The faculty of that medical college at the time of chartering consisted of one physician (a graduate), his wife, and his brother, who was not a graduate of medicine. This charter was carried back and they established a medical college. They have been advertising in the daily press, in the Sunday papers, and are 'graduating' people all over this State today. I am glad to say, however, that the matter was brought to the attention of the Attorney-General, and after a large amount of correspondence, and by accumulating sufficient evidence, he sued them in San Antonio to revoke their charter. The West Texas Medical Association called a meeting and investigated the matter, and took up a collection of $50.00, the amount charged for a diploma, and selected a negro porter in San Antonio to enter the school and get his diploma. He entered the school, attended seventeen days, and upon payment of $50.00 he received a diploma signed by ten or fifteen 'members of the faculty,' all of whom I suppose are 'graduates' of the same school, except the one man who founded the college originally. I think our Attorney-General deserves great credit for his efforts in trying to break up this school, with the laws as they exist in this State today. But it only goes to show what the State of Texas is doing in the way of medical education. We have upon the one hand the Medical Department of the University of Texas, which requires an entrance examination before young men can go there and take up the study of medicine, and requires a four years' rigid course, with every facility for teaching medicine in all of its branches. Upon the other hand we have the same State issuing a charter to an institution like the one in San Antonio, in which they graduate ignorant porters in seventeen days. I say this is a fine state of affairs in the State of Texas. I want this Medical Association to speak in no uncertain tones, and I would like to suggest, that inasmuch as we have over $1100 in our treasury, this meeting appoint a committee to consist of members of this Association outside of Austin (and I say outside of Austin because the physicians there will be bound to look after their patients more or less), to go to the next meeting of the Legislature and act as a lobbying committee. Let them go there and meet the different members of the Legislature and show these facts to them, and explain to them why it is so important for us to have laws regulating the practice of medicine in this State, not for the protection of the physician, because he is cognizant of these matters and he and his family are safe, but for the people all over this State, who go in the dark hours of the night for a physician to come and see a member of their family, who might be critically ill, thinking him prepared to practice his profession because his diploma was registered according to the laws of this State. Such a committee should be sent there; but we should do more. We should go out as individuals in the different walks of life, show these facts to the people all over the country, and we should see our different members of the Legislature and Senate, from this time on, before the election and after the election, and sit down and reason with them and show them the foolishness of the present methods regulating the practice of medicine in this State. A great many of the legislators are intelligent men, and are anxious to do right, but they are entirely ignorant of what the condition of affairs are in this State, and as they have so much to do, when they get to the Legislature, with two or three hundred bills having been introduced, that it is impossible for them to give the attention to the subject as its importance deserves. Therefore, I suggest we commence our campaign of education at once. And while upon this point, I desire to say that our prospects for medical legislation are brighter today than they have ever been in the State of Texas. This college affair at San Antonio will be published and talked of all over the State, and in addition we have this assurance: Several of us called upon the Governor recently, when we learned of the existence of this college at San Antonio. Dr. Wilson, our ex-president, I believe was present upon one occasion. The Governor assured us that in case the Attorney-General could not cope with the affair that he would send in a special message at the called session of the Legislature then in session. After debating over the subject we decided the called session of the Legislature already had more to do than it could possibly accomplish. Therefore, we made this proposition to the Governor: That in case he was our Governor next year if he would promise us to recommend in his general message to the Legislature a revision of our health laws, or something to cover just such cases, we would do what we could with the present law until that time, and he assured us he would make such a recommendation. Therefore, gentlemen, with such an affair as this San Antonio school known and understood over the country, with the promise of the Governor to recommend additional medical legislation in his message, and if the members of the medical profession will go out and educate the people all over this State, we ought to accomplish some good results. I think there is but one more thing necessary; and that is for the members of this Association, so far as they can, to go as delegates to the next democratic nominating convention which meets in Waco in June; let as many as can possibly do so meet here in June in that convention, hold a caucus, see what their voting strength is, and demand that one member of the profession at least be placed on the platform committee, and let that member stand up and fight for the rights of the people in the way of medical legislation. I believe that with such a recommendation placed in our platform, coupled with others mentioned, at the next session of the Legislature we will get some medical legislation.

"Again, I think there should be some law regulating the practice of midwifery in this State, for at the present time we have all over this State women, both white and black, who are permitted to practice midwifery, who are unqualified to attend women in confinement—that most critical period in a woman's life. These people in the majority of instances are entirely ignorant. They have not the least conception of what they are doing, except in a very vague and general way. They know nothing at all about meeting any emergency that might arise. They know nothing about the protection of the eyes of the new-born, which would reduce the cases of blindness in our own blind institution in a large measure. They know nothing at all about the resuscitation of the infant born lifeless, when, in most cases, its life can be saved. I say that we should stand up and make a demand that this State offer some protection to the mothers of this country.

"I have but one thing more to say in these remarks, which are scattered at random, and that is, we should demand some legislation concerning criminal abortions, and we should stand up as a body of physicians and try, if possible, to prevent the present condition of affairs in this State which deals with criminal abortion. I believe the time is upon us when the State of Texas should make some effort to protect the lives of its unborn children as well as those living. I am sorry to say, gentlemen, we have physicians in the State of Texas who follow such work as a business, for the small sum of $15.00, $25.00, or $50.00; physicians who will murder an innocent infant in its mother's womb, placed there for its protection and life, for money. I believe we should combine, put on a bold front, and feel that whenever we can do anything to show up such cases and do not, that we are ourselves *particeps criminis.*

"I feel this way, gentlemen, in such cases. Whenever a person comes into my office and makes such a request I feel that I stand before the Judgment Bar, and that if I can say anything in pleading for the life of an unborn human being who is not able to defend itself, I should do so. And I make it a rule, without any exception, irrespective of the person making the request, to stand up and speak out, *and speak out plainly,* and I shall do it as long as I live. I hope the medical profession all over this State will do the same thing. We should recommend and ask for some legislation looking into this matter. We should condemn in no uncertain terms the publication in our daily press (we see it daily in almost all our leading dailies) of the advertisement of articles sold for no other purpose than to produce abortion, and such advertisements are run for the small sum of fifty cents or a dollar, by the publishers of those papers. We should speak of these things, and we should in some manner try to eradicate them from the face of the earth.

"If I were an artist and could paint a picture I would select as my subject the horrible face of the criminal abortionist; I would make him the central figure of an immense picture, and all around him I would have the beautiful innocent faces of little children pointing at him and condemning him for his horrible deeds." (Applause.)

### DISCUSSION.

DR. F. E. DANIEL (appointed to "open the discussion") said: I would say, first, in reference to the only "health" law we have on our statute book, the one to prevent the adulteration of foods and drugs and provide for food inspection, that it was drawn with a great deal of care and passed the Legislature, but the Legislature failed to make any appropriation to carry out its provisions, and it remains a dead letter. We have no "health" laws upon our statute books now. Quarantine laws cannot be called health laws.

We are all agreed that evils exist and that reform is necessary. I do not think there is any one in this room who will not agree with the proposition that the eleemosynary institutions of this State should be taken out of politics, but how are you going to do it? That is a question that this Association has been wrestling with for twenty years. Dr. Massey suggests that we should educate the people, and says that we should not hold the Legislature responsible. Again I ask you, how are you going to educate them? You can take a horse to the water, but you cannot make him drink. You may send Dr. West's paper, and Dr. Smith's paper, and Dr. Harrison's splendid report to every citizen of the State of Texas, in a sealed envelope, with a two-cent stamp on it, but there is no power on earth that can make them read it. The people may be primarily responsible, but in my judgment the evil underlying this whole thing is our system of legislation. The State of Texas pays a legislator a laborer's wages and expects to command law-making talent. Of course, there are exceptions; but you all know that for the most part those who go to Austin to make our laws are not only not educated in the question of State medicine and public hygiene, but they are educated in very little else. I have seen members there who tell me frankly they know nothing of the subject, and give me to understand that they don't want to know. Now, what are we going to do, so long as our national government remains without any central organization? The only organization that the United States has ever had on the great question of public health was the National Board of Health, which had to give way under political pressure and has passed into a quarantine bureau.

As showing the value of sanitation as a life-saving institution, we quote the following from Professor Andrew D. White's great work, "*The Warfare of Science with Theology*": "The recent history of sanitation in all civilized countries shows triumphs which might well fill us with wonder, did there not rise within us far greater wonder that they were so long delayed. Amazing is it to see how near the world has come again and again to discovering the key to the cause and cure of pestilence. *It is now a matter of the simplest elementary knowledge that some of the worst epidemics are conveyed in water.* But this fact seems to have been discovered many times in human history. In the Peloponnesian war the Athenians asserted that their enemies had poisoned their cisterns; in the Middle Ages the people generally declared that the Jews had poisoned their wells; and as late as the cholera of 1832 the Parisian mob insisted that the water-carriers who distributed water for drinking purposes from the Seine, *polluted as it was by sewage,* had poisoned it, and in some cases murdered them on this charge. Diseases like typhoid fever, diphtheria, pulmonary consumption, scarlet fever, pneumonia, and *la grippe*, which now carry off so many most precious lives, should have long since ceased to scourge the world."

Yet the United States government has permitted three or four millions of people in Chicago to turn their sewage into the Mississippi river for the inhabitants along the banks of that stream to drink and be poisoned and suffer and die. It is a shame and outrage, but what are we going to do? If any gentleman present can suggest any remedy I would be glad to have him do it. I have despaired of ever impressing the members of the Legislature with any conception of public hygiene or State medicine. I made the remark just now that there are very few of our public men, statesmen, even governors, who understand the meaning of the term. There are still fewer who comprehend it in its entirety, and I am also satisfied there are a great many physicians who have but a very vague conception of the term "State medicine." I hope I will be pardoned for reading very briefly the best definition on the subject I have ever seen. In the last few days I have seen a work gotten up to send to the Paris Exposition to show "the wonderful progress of sanitation in this country." It contains and I have copied here Dr. Billings' definition. I believe that our congressmen, our governors, our statesmen, our most enlightened public men do not understand the question, or know the meaning of it, or grasp its extensive bearing upon the public health. I read the paper:

HYGIENE—STATE MEDICINE.

Billings, in Buck's *Treatise on Hygiene and Public Health*, says: "In its broader sense, the study of hygiene includes the examination of the conditions which affect the generation, development, growth and decay of individuals, of nations, and of races—being, on its scientific side, coextensive with biology in its broader sense, including sociology, rather than with physiology merely, as some writers state."

"The term 'State Medicine' is a broader term than public hygiene, since the former includes the latter, together with legal medicine, medical education, and all subjects which treat of the relation of the physician to the State."—[Dr. S. W. Abbott, Secretary Massachusetts State Board of Health, in "*Monographs on Social Economics*" prepared for the U. S. Commission to the Paris Exposition, 1900.]

Billings adds: "* * * The direct pecuniary loss to this country on account of preventable sickness and mortality is certainly over $100,000,000 annually, without taking into account expenditures incurred on account of sickness, etc., or the unusual losses due to great epidemics, both from waste of life and injury to commerce. * * * It is evident, therefore, that hygiene is not only a subject of scientific interest to the student, or to medical men, but that to the political economist and to the legislator its problems and discoveries ought to be of great practical importance—greater, in fact, than many of the subjects with which these gentlemen usually occupy themselves."

There is a table in the book referred to showing the amount of money expended in each of the United States for sanitary purposes. Texas is credited with an expenditure of two cents per capita—second only to Massachusetts and Florida. There is not a dollar expended or appropriated in Texas for any sanitary purpose except for quarantine. It goes to keep out diseases of foreign origin, which, if properly managed, ought never to have been permitted to get out of the place where originated. My idea of quarantine is that our system should be reversed. Bubonic plague, yellow fever, smallpox should not be permitted to get out from where it arises. Suppose there should be a band of robbers and murderers loose in the streets of Waco, and instead of locking them up, every citizen should go home and bar his doors. That is a simile that fits our quarantine.

If State Medicine were understood by our lawmakers, our officers, our governors, and its principles were applied, not only many evils would be remedied, but such shams as this "Medical College" in San Antonio would not be possible. I mean by that to say that this Association in its efforts before the Legislature time and again has failed to impress governor or lawmakers with the fact that there is a *difference between a license and a diploma!* The law recognizes no difference. Hence such abuses as this fake college become possible. The holder of one of their diplomas, who has it recorded in the county in which he practices, can practice, and there is no law to prevent him. If the principles of State Medicine were understood all of these evils could be easily reached. Now, how are you going to "educate the people?" The newspapers in this State will not publish any sanitary or medical article unless paid to do so. They pay no attention to the subject. They throw it into the wastebasket.

I believe our efforts should be directed primarily to the creation of a great central organization at Washington. If that is not done I see very little hope of accomplishing any great reforms in this State. The doctor speaks of "demanding this legislation." You may plead; you may beg;— but "demands" are out of order. We have utterly failed to make these gentlemen understand that the protection of the lives of the people of Texas comes within the scope of the police powers of the State. It is one of those rights that has never been delegated to the Federal government. We have insisted that the State should appreciate that, and should put such restriction around the privileges of practicing medicine as will rob it of its dangers. I believe that if our public men had any conception of this subject they would not fail to be impressed with its vast importance. They have not studied it. Those who go to the Legislature have ambitions; they have political aspirations; they have something of more importance to themselves to attend to. They have not had time to study much of anything, they are so young, some of them.

Dr. Rosser: Dr. Daniel in his remarks said that if any man knew how to bring about the desired reform he would like to hear from him. I do not rise as a Moses to lead this congregation out of the wilderness, but I believe the suggestion made this morning by Dr. Wilson in his report the most sensible one I have heard in many a year relative to any effort regarding legislation. We have got to get away from sentiment and get down to business. You know in every community if a man has any sort of idea about politics, and undertakes to help shape the policies of his community, he is considered a politician, and his enemies are at once put to work talking to his disparagement. None of us are politicians, and none of us are going to be politicians, but there is a way to attend to just this business. That is, not to talk to men who are already elected and petition them to do something when they are not interested in it, but the way is to influence the selection of a man who can influence a political body. You understand the leverage which a Governor has. He has committees to appoint. He has this office and that office to give out. When he gets there and begins to formulate his committees he can say to this fellow from this other county: "Here is a certain measure going to come up. How do you stand on this?" The Governor does not mean, perhaps, that if he does not line up with him on his policies he will not appoint his man, but he wants to hear from the fellow first. Now, the Governor can do these things. We have no hope of the present Governor doing anything about this matter. He is already elected. He is going to be elected next time without opposition. He is independent of us now. Two years from now is as early as we can hope to do anything unless he wants to do it. But this campaign committee Dr. Wilson spoke of this morning, handled intelligently, can do something. If three or four men want a nomination, which means an election, those men want the influence of the people to get the nomination. As a matter of course every community has a doctor, and at the crossroads we recognize perfectly well there are just about three men who have considerable influence there. One is the doctor, one is the school teacher, and the other is the preacher. Those people can get together and control the community pretty largely on anything they want to talk about. If there is a campaign committee, and that campaign committee means business, and these candidates for office are interviewed on these subjects that we are interested in we can do something. The moment a man steps up and says he wants to be Governor have that campaign committee call on the different members of the profession to write to him and say to him, "How do you stand on the subject of medical legislation?" You will find then he will become interested in it, and when he gets on the stump have some men ask him how he stands on it and get him to commit himself, and when the primary comes you vote for the fellow that is for you. When you get so you are taking more interest in this medical legislation than you are in some other little matter, or in some personal friendship, then you will accomplish something. I believe it can be done as surely as any other thing can be done, because it is the most important thing that can be discussed. You can make it so that no man will make a canvass in this State for Governor who will not advocate it in his speeches. Of course, the legislator, when he gets up there, has read what the Governor says, because the Governor has the power to make or unmake him. He has ambition. He will do what the Governor wishes. I think this is the course to pursue, and it must be done practically and not on sentiment.

Dr. Saunders: In order that the profession may know something about these diplomas I would ask you to go to the records in the county clerk's office and see what diplomas are recorded there. Being interested in that, I took occasion to find out how many diplomas were registered in Tarrant county from bogus medical colleges. One hundred and fifteen were registered from one bogus medical college in the city of Chicago. If that many were registered in Tarrant county, how many are registered in Dallas county, and Bexar county, and some other counties? Just look into this question.

Dr. Wilson: Just one word, and that for the benefit of Dr. Rosser. I will state that two months ago Dr. Denton, of Austin, and myself called on the Governor on this subject, and the Governor made us a faithful promise that if he were re-elected next fall that in his next message, if we would write to him upon the subject, he would embody this matter and urge it strenuously. He made us a faithful promise, and, therefore, we need not wait for two years. But one other point, and that is as follows: Talk is very cheap, and we all know what we need in the State, and everybody knows just how it should be remedied, and can get up here and tell all about it, but when we leave these halls we forget all about it. You appoint a committee and that is the last you hear from them in nine cases out of ten. They go home and they forget all about it, and probably when the next Legislature meets they are asked about it, and they will say they have forgotten it. They do not do anything. The proper way is for your committees to go to work at once, and organize and appoint sub-organizations, and get them to work, and urge them to work, and let it be done immediately and not wait. If you do that you can get something done, but if it is delayed until it is too late it is useless. I do not believe in delaying the matter as Dr. Rosser says, but go to work on the next Legislature. Get the Governor to help you as he has promised to do, and I believe you can do something.

Dr. Massey: The gentleman who has just taken his seat argues on the line I was arguing a while ago. I say begin your campaign of education as soon as you get home. Do not wait until the next Legislature meets. Begin your campaign of education at once, and then you may hope to accomplish something. These gentlemen have all argued in favor of the campaign of education. You will find no one who is going to be willing to vote to make a new law, or to change a law, until he believes his constituency is in favor of it. Dr. Daniel has mentioned that he has spoken to different legislators and they tell him they don't know anything about such things and don't want to know about such things. You ask him what he knows about the wishes of his constituency. They are not ignoramuses who go to the Legislature. They may not be orators, and they may not be the brightest men in the world, who will set it afire, but they know what their constituents want in the way of legislation, and as a rule they represent the wishes of their constituency. This talk has wandered very far, though, from the address of Dr. Smith. We were supposed to be discussing the very able address the doctor delivered, and in many respects I consider it one of the most able that has been presented. But there are one or two things on which I beg leave to differ from him, and there was one thing on which I think the doctor exhibited a lamentable weakness, and that was about the bogus college at San Antonio. The whole society made a great hue and cry. The Attorney-General has finally become interested and he will close up the institution, but in order to do this the West Texas Medical Association went so far as to graduate a negro doctor, and he is a doctor in this State as well as you or I, and he enjoys the same privileges and rights that you and I enjoy. He can practice as a doctor today as well as you can. I wish to relate an instance of the same kind that occurred in Houston, I think in the year 1893. There was a medical college got a charter from the Governor of this State, the same as the San Antonio institution. It was just as good. They located within about four blocks of where I lived. Very few doctors knew the institution existed. Accidentally finding it out I went before a justice of the peace and had warrants issued for the three men interested, and two of them were arrested, and they were put under $200 bond, and after a while they skipped the bond. That college was broken up in Houston in twenty-four hours time, without a hue and cry and without graduating a negro doctor. That institution also had a charter from the State, issued, I think, by Allison Mayfield, Secretary of State at the time. I doubt very much whether Dr. Red or Dr. Scott knew anything about the matter at all. It was mentioned in the paper that the proprietors and instigators had been arrested and placed under bond to await the action of the grand jury. Let me urge one other thing, gentlemen. Instead of appointing a large lobbying committee to go to Austin and attempt to lobby with the members of the Legislature already elected, go home and begin your campaign of education. You have some funds on hand. A committee might be appointed to begin the agitation of this question through the public press. It is stated the public press will not print anything of this kind. You spend a little of the $1400 of $1500 you have with the public press, and they will print your articles. Let your legislative committee begin this kind of work through the public press at once. There is one method that has been urged by Dr. Harrison. I hope the doctor will give us a talk on the subject and explain his methods a little more fully.

Dr. Daniel: Dr. Massey is mistaken about our action against the San Antonio college. It is not shut up yet. The Attorney-General has taken hold of it. We asked him how it could be gotten at. He said: "If you gentlemen can furnish the facts that is all I want. I will do the rest." He has been furnished with the facts, and when that establishment is closed up it will be in due process of law, and not frightened out of the State by a threat.

Dr. Harrison: It is one of the first duties of a State to protect the lives and property of its people, whether it be from the practices of a medical mountebank or the dagger of an assassin. The duty is of the same magnitude, and the laws for which we have been asking are of equal importance for protection against the one or the other. Twenty-five years ago I submitted a bill to the Legislature of the State of Texas, providing for the establishment of a Board of Health and Vital Statistics. That bill carried through the lower house and got to a second reading in the Senate of the State of Texas, and that body adjourned before it got the third reading in the Senate or it would have been a law. Since that time one interest or another has defeated legislative action on this subject, until at the last session of the Legislature the matter was submitted by a committee from this Association, and it was urged with all of the ability that a number of the best men in this body could command before that legislative body, but for some cause or other it was not passed. The committee which had charge of that subject was continued by the last session of the State Medical Association, and is still on duty, and proposes to continue its labors as long as it receives the sanction of the Texas State Medical Association. Why the subject was not acted on by the Legislature, why it was impossible to secure a report on the bills that were submitted, involving so much for the weal or woe of the State, is more than I can conceive of to save my life. Prior to the election our Governor had pledged himself to give the matter his earnest attention. After his inauguration he gave it the cold shoulder, and it was utterly impossible to get his attention to it by any effort the committee was able to make. As the matter stands now, there has been before the State Medical Association and before the State at large, for a period of two years, this proposition to give protection to the public health by means of a State Board of Health, from internal as well as external disease. The gentlemen who have preceded me have told me what should be done. They have not told me yet how to do it. The proposition to establish a Board of Health was submitted fairly and squarely to the Governor, and it met his sanction before the election, but after his election he repudiated it. What the result will be of our present action I cannot well conjecture. I believe, however, that we should tackle the subject as the late session of the Colorado Medical Association did.

This, gentlemen, represents the sentiment that I have been trying to inculcate, and what I hope you gentlemen of the Association will try to inculcate at your homes between now and the next legislative election.

Dr. Jones, of Austin: I had not thought to participate in this discussion, inasmuch as I am connected with the quarantine department of the State, and I do not now rise to say anything in connection with that department. But I am now, and have been for years, ever since I was connected with the medical profession, vitally interested and practically interested, in all proper medical legislation, such as would tend to elevate the profession and tend to protect the public health. Now, I shall not say, as my friend Dr. Rosser says, that I am not a politician, because you would not believe me if I did; but from the standpoint of a politician, I want to make a remark or two. I want to say that there is but one man in the world that can make friends by abusing people, and that is the traveling evangelist. You have got to rub the hair the right way and you need not start out in any other way to do it. If you start out with the proposition that the Governor and the Legislature from some occult reason are not going to do what you want, and they are to blame for it, they are not going to do it. Dr. Harrison has drawn a bill which was presented to the last Legislature, but was submitted too late for any legislation to pass what was submitted at that time. That bill can be passed, I want to tell you, on the reputation of a politician, but if you start out to reform everything in Texas you will not get it through, because it will be too radical and the Legislature will not be prepared for it, and they will want to hear from home first. I am in sympathy, I am glad to tell you, with every legislative action that looks to the protection of the public health. Our quarantine law in ineffective. Nobody knows that so well as the man who has tried to enforce it. We all admit it. You can get the quarantine law amended, because I do not suppose anybody contemplates absolutely abolishing any kind of quarantine, because the most enlightened States have it. You can get the quarantine law amended. You can get the State Board of Health and Vital Statistics, and I think if you do not try to get it all at one time you could get a law passed to control the license to practice medicine. But there is another thing. I do not sympathize with this attempt here—(I know it was not so intended, but it has that effect)—to blacken the reputation of our State on this subject. I do not believe there is another State south of the Mason and Dixon line that spends as much money for public health purposes as Texas does. It may be unwisely spent, but it is spending money. It may be misdirected; it may be badly spent, but I do not hear of our profession getting around in a practical way in their neighborhood and advising ways and means to spend it better. Reforms should frequently begin at home. Your next door neighbor needs reform in his back door premises, very likely. Do you get out and get him to do it? That is practical sanitation. I am not here to speak for the Governor or the State Health Officer, but I will speak for myself, that I have been intimately acquainted with the last four Legislatures that have met at Austin. I have had business with them. I know their sentiment on this question, and the State Medical Association has come up there and asked so many diverse things, and not agreeing among themselves as to what it actually did want, that the Legislature would have had a hard time pleasing the State Medical Association, much less the people of Texas at large. Take some simple, direct proposition, and put it through, and when you get that done take up the next reform, and we will soon have it.

Dr. Shropshire: I have talked with our legislators, the Senator and Representative from my district, and they say: "We are willing to do all we can for you doctors." The legislators think that we require some legislation for ourselves, not for a moment entertaining the idea that we want the legislation for the protection of the community. I just want to correct the impression that we want the legislation for ourselves, and that we do not seek the legislation for the benefit of the community.

Dr. Smith: I shall say but a very few words. I desire, however, to say something more in addition to what Dr. Daniel said in reply to Dr. Massey, of Houston. We had looked up the question of how we should proceed to get rid of that school at San Antonio. We appeared before the Attorney-General. We showed the Attorney-General what had been done as regards the college that the doctor referred to. He told us that he could not act unless he had more data, and he advised the graduation of some one from that college, and upon that advice the Western Texas Medical Association graduated this negro, to which I referred. They were acting under the guidance and at the suggestion of the Attorney-General, and I believe they were doing right. As to the publication of anything concerning these schools in the public press, I will say, that the article that first appeared in the *Texas Medical News* I sent a marked copy of it to the editors of the daily papers in the State, and wrote them a personal letter urging upon them the importance of saying something editorially about this school, and showing the necessity of the State being aroused to take some steps to suppress such injustice. I had two or three letters from the editors, one in particular from the *Galveston News*, and one from the *Houston Post*. They said that they had consulted their attorneys and were advised to publish nothing concerning the school; that we had in the State of Texas a treacherous libel law, and that large daily papers could not afford to publish anything of the kind. Therefore, I think Dr. Massey is mistaken. I appreciate the able discussion given the few crude remarks that I have made, and I hope the profession will take on renewed energy and push this educational work all over the State, and I believe we will accomplish something at the next session of the Legislature.

